# Comparison of Various Easy-to-Use Procedures for Extraction of Phenols from Apricot Fruits

**DOI:** 10.3390/molecules16042914

**Published:** 2011-04-04

**Authors:** Ondrej Zitka, Jiri Sochor, Otakar Rop, Sylvie Skalickova, Pavlina Sobrova, Josef Zehnalek, Miroslava Beklova, Boris Krska, Vojtech Adam, Rene Kizek

**Affiliations:** 1 Department of Chemistry and Biochemistry, Faculty of Agronomy, Mendel University in Brno, Zemedelska 1, CZ-613 00 Brno, Czech Republic; 2 Department of Food Technology and Microbiology, Faculty of Technology, Tomas Bata University in Zlin, Namesti T. G. Masaryka 275, CZ-762 72 Zlin, Czech Republic; 3 Department of Veterinary Ecology and Environmental Protection, University of Veterinary and Pharmaceutical Sciences, Palackeho 1-3, CZ-61242 Brno, Czech Republic; 4 Department of Fruit Growing, Faculty of Horticulture, Mendel University in Brno, Zemedelska 1, CZ-613 00 Brno, Czech Republic

**Keywords:** polyphenols, apricot, high performance liquid chromatography, CoulArray electrochemical detector, UV-VIS detector

## Abstract

Phenols are broadly distributed in the plant kingdom and are the most abundant secondary metabolites of plants. Plant polyphenols have drawn increasing attention due to their potential antioxidant properties and their marked effects in the prevention of various oxidative stress associated diseases such as cancer. The objective of this study was to investigate a suitable method for determination of protocatechuic acid, 4-aminobenzoic acid, chlorogenic acid, caffeic acid, vanillin, *p*-coumaric acid, rutin, ferulic acid, quercetin, resveratrol and quercitrin from apricot samples. A high-performance liquid chromatograph with electrochemical and UV detectors was used. The method was optimized in respect to both the separation selectivity of individual phenolic compounds and the maximum sensitivity with the electrochemical detection. The lowest limits of detection (3 S/N) using UV detection were estimated for ferulic acid (3 µM), quercitrin (4 µM) and quercetin (4 µM). Using electrochemical detection values of 27 nM, 40 nM and 37 nM were achieved for ferulic acid, quercitrin and quercetin, respectively. It follows from the acquired results that the coulometric detection under a universal potential of 600 mV is more suitable and sensitive for polyphenols determination than UV detection at a universal wavelength of 260 nm. Subsequently, we tested the influence of solvent composition, vortexing and sonication on separation efficiency. Our results showed that a combination of water, acetone and methanol in 20:20:60 ratio was the most effective for *p*-aminobenzoic acid, chlorgenic acid, caffeic acid, protocatechuic acid, ferulic acid, rutin, resveratrol and quercetin, in comparison with other solvents. On the other hand, vortexing at 4 °C produced the highest yield. Moreover, we tested the contents of individual polyphenols in the apricot cultivars Mamaria, Mold and LE-1075. The major phenolic compounds were chlorgenic acid and rutin. Chlorgenic acid was found in amounts of 2,302 mg/100 g in cultivar LE-1075, 546 mg/100 g in cultivar Mamaria and 129 mg/100 g in cultivar Mold. Generally, the cultivar LE-1075 produced the highest polyphenol content values, contrary to Mold, which compared to cultivar LE-1075 was quite poor from the point of view of the phenolics content.

## 1. Introduction

**Figure 1:**
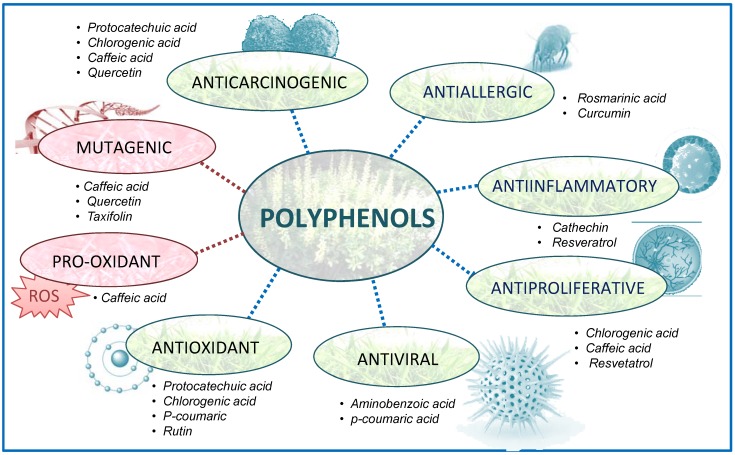
Polyphenols and their biological properties. Polyphenols have a great variety of beneficial effects like anticarcinogenic (e.g., quercetin, protocatechuic acid) antiallergic (e.g., rosmarinic acid, curcumin), antiinflammatory (e.g., cathechin, resveratrol), antiproliferative (e.g., chlorgenic acid, caffeic acid, resveratrol), antiviral (e.g., aminobenzoic acid, p-coumaric) and antioxidant (e.g., rutin, chlorgenic acid, quercetin on human health. Their antioxidant properties and abilities to modulate several enzymes are also important. Some flavonoids have also mutagenic (e.g., quercetin) and/or prooxidant effects (e.g., caffeic acid) and they may interfere with essential biochemical pathways.

Nowadays the lifestyle trend is to consume a diet rich in fresh fruit and vegetables beneficial to our health. Fresh products are rich in various nutrients such as fiber, vitamins, minerals, organic substances and finally phenolic compounds. Phenolic compounds are a large class of naturally occurring compounds generally synthesized via the shikimate pathway. Another pathway, the polyketide pathway, can also provide some phenols, e.g., orcinols and quinones. Phenolic compounds derived from both pathways are quite common, e.g., flavonoids, stilbenes, pyrones and xanthones [1]. Polyphenols are widely present in plants have a wide range of functions [2,3,4]. They protect them from oxidative stress, UV light, pathogens, eating by herbivores, lignans form a mechanical reinforcement of the plant body and others can play a role as cell signalling molecules [5,6,7,8]. In humans and animals they prevent common diseases, including coronary diseases, cancer, neurodegenerative diseases, gastrointestinal disorders and others [9]. Other authors have described how polyphenols affect endothelial function and as a consequence, blood pressure [10]. A basic scheme of polyphenols properties is shown in Figure 1. This is the reason that polyphenols have attracted great interest in the functional foods, nutraceutical and pharmaceutical industries. They are used as aromatic, taste-supplying and colouring substances, although the unpleasant taste of most phenolic compounds limits their applications [11]. On the other hand, the toxicity of simple phenols was demonstrated [12,13]. Experimental studies of the "extended toxicity" of substituted phenols are mainly of two types: the toxicity due to phenoxyl radical formation and the toxicity caused by metabolites, for example, the formation of quinones [14]. Quinones also play a major role in allergic contact dermatitis caused by plants. The principal allergens are benzoquinones or naphthoquinones but also compounds, such as catechols and other phenolic or flavonoid compounds, which are bioconverted into *ortho*-quinones or *para*-quinones [15].

### 1.1. Representative polyphenols

#### 1.1.1. Protocatechuic acid (3,4-dihydroxybenzoic acid, PCA)

Protocatechuic acid (PCA) present in natural products (in plants and fruits), is also a catabolite of epinephrine. The efficiency of PCA as a strong anti-radical, antioxidant and moderate scavenger of H_2_O_2_, has been widely reported. Its free-radical scavenging ability inhibits chemical carcinogenesis and protects against hydroperoxide-induced toxicity [16,17]. Many studies based on animal experiments have shown that PCA has strong anti-cancer effects [18,19]. In addition strong neuroprotective effects have been demonstrated [20]. 

#### 1.1.2. 4-Aminobenzoic acid (PABA)

PABA is a cyclic amino acid belonging to the vitamin B group and is used as a protective drug against solar insolation and in diagnostic tests for the state of the gastrointestinal tract in medicine [21]. PABA occurrence in plant and animal tissues have been determined [22]. PABA is an inducer of endogenous interferon and immunomodulator and displays a virucidal, synergistic antiviral effect when combined with chemical drugs and the properties of a direct anticoagulant [21]. Other studies described anti-virulent properties and therapeutic effect against typhoid and rickettsias diseases [23].

#### 1.1.3. Chlorogenic acid [3-(3,4-dihydroxycinnamoyl)quinate]

Chlorogenic acid is structurally an ester of polyphenolic caffeic acid and cyclitol L-quinic acid [24]. It is an important intermediate in lignin biosynthesis [25]. It is also an antioxidant [26] and can contribute to treatment of atherosclerosis and ischemic reperfusion illnesses [27]. Other authors have described the properties of chlorogenic acid as an inhibitor of tumour promoting activity [28]. Moreover, chlorogenic acid decreases the cholesterol level in the blood of alcoholics by stimulation of secretion of bile acids [29].

#### 1.1.4. Caffeic acid [3-(3,4-dihydroxyphenyl 2-propenoic acid]

Caffeic acid is a key intermediate in the biosynthesis of lignin, one of the principal sources of biomass. It presents antioxidant effects *in vitro* and might therefore contribute to the prevention of cardiovascular disease [30]. Caffeic acid is a hydroxycinnamic acid derivative that is widely distributed in plant-derived food products [24]. Studies indicate that some dietary compounds may have concentration-dependent antioxidant or pro-oxidant activities [31]. Caffeic acid also inhibits the production of carcinogenic and mutagenic N-nitrosation compounds [32] and protects from DNA damages *in vitro* [33,34].

#### 1.1.5. Vanillin (4-hydroxy-3-methoxybenzaldehyde)

Vanillin is most prominent as the principal flavour and aroma compound in vanilla that is abundantly used in the food industry. Vanillin decreases the incidence of large intestinal carcinomas [35]. Vanillin also considerably suppresses bacterial formation [36]. The ability of vanillin inhibiting photosensitization-induced single-strand breaks in plasmid pBR322 DNA has been examined in an *in vitro* system, independent of DNA repair/replication processes [37]. Therefore, interaction of singlet oxygen with vanillin was investigated [37,38].

#### 1.1.6. *p*-Coumaric acid (3-(4-hydroxyphenyl)-2-propenoic acid)

Coumaric acid is a hydroxy derivative of cinnamic acid [39]. *p*-Coumaric acid has antioxidant properties and in peroxidising lipid systems mediated by metamyoglobin [40]. Moreover, the antioxidant effects of LDL cholesterol have been demonstrated so it also prevents atherosclerosis [41]. It is believed to reduce the risk of stomach cancer by reducing the formation of carcinogenic nitrosamines [40].

#### 1.1.7. Rutin 

Rutin is an antioxidant with many interesting pharmacological effects. There is an evidence that rutin protects plants against UV radiation by activation of enzymes of the phenylpropanoid pathway [42,43]. Rutin exerts renal protective effects, probably by inhibiting ROS and antioxidant activities [44]. Positive effects of rutin on human health are primarily in decreasing of blood pressure [45], decreasing of permeability of blood vessels and swelling creation and thus prevent atherosclerosis [46].

#### 1.1.8. Ferulic acid [(*E*)-3-(4-hydroxy-3-methoxyphenyl)prop-2-enoic acid]

Ferulic acid (FA), like many phenols, has concentration-dependent antioxidant effects in terms of inhibition of lipid peroxidation and reactive oxygen species [31]. Moreover, the photoprotective properties of FA on human keratinocytes help to prevent damage by ultraviolet (UV) radiation and skin carcinogenesis, have been described [47,48]. The effects of FA on the proliferation of neural stem/progenitor cells (NSC/NPCs) *in vitro* and *in vivo* was investigated [49].

#### 1.1.9. Quercetin

Quercetin is a dietary polyphenolic compound with potentially beneficial effects on health [50]. Numerous studies confirmed quercetin’s properties as an antioxidant [51,52]. Quercetin can quench reactive oxygen species and protect the organism from pro-oxidative damage [53]. Quercetin also protects the organism against coronary diseases [54,55], lung cancer and asthma [56]. An *in vitro* study investigated the possible radioprotective effects of the natural substances propolis and quercetin on gamma-irradiated human white blood cells [57].

#### 1.1.10. Resveratrol 

In recent years, resveratrol has become a popular nutritional supplement used by humans all over the world. Detailed research has been conducted to determine the efficacy of its use both in preventive and therapeutic dimensions [58]. Resveratrol, with its known potency and wide variety of health benefits, has shown promising results in minimizing cardiovascular complications [59] including hypertension [60] and it reduces oxidative organ damage in the renal system [61], hypertrophy [62], ischemic heart disease and atherosclerosis [63]. However, a great deal of controversy exists regarding the use of resveratrol as an anti-aging compound [64].

#### 1.1.11. Quercitrin

Quercitrin is a glycoside formed from the flavonoid quercetin and the deoxy sugar rhamnose. It is a constituent of the dye quercitron. It is widely distributed in many edible (Qi 1) [65] and medicinal (Qi 2) plants [66], and exhibits a wide range of biological activities, such as antioxidant [67], inhibition of acetylcholinesterase [66] and serine/threonine kinase pim-2 [68], hepatoprotection [69], and antimalarial activities [70]. Recently, the anti-HIV and cytotoxic activities [71] against human oral tumour cell lines were reported.

### 1.2. Extraction

The extraction of phenolic compounds is one of the stages in the sample preparation process [72]. Numerous methods such as microwave, ultrasound-assisted extractions, and techniques based on use of compressed fluids such as subcritical water extraction (SWE), supercritical fluid extraction (SFE), pressurized fluid extraction (PFE) or accelerated solvent extraction (ASE) as extracting agents, have been developed in recent years for the extraction of phenolic compounds from plant materials [72]. The effect of extraction conditions on polyphenols has been a contentious issue, particularly when comparing different raw materials. Many factors such as chemical nature, solvent composition, extraction time, extraction temperature, and solvent to solid ratio may significantly influence the extraction efficacy and yield [14]. The chemical nature of plant phenols vary from simple to highly polymerized substances that include varying proportions of phenolic acids, phenylpropanoids, anthocyanins and tannins, among others. They may also exist as complexes with carbohydrates, proteins and other plant components; some high-molecular-weight phenols and their complexes may be quite insoluble. Therefore, phenolic extracts of plant materials are always a mixture of different classes of phenols that are soluble in the solvent system used. Additional steps may be required for the removal of unwanted phenolics and non-phenolic substances such as waxes, fats, terpenes and chlorophylls [73,74]. Solid phase extraction (SPE) techniques and fractionation based on acidity are commonly used to remove unwanted phenolics and non-phenolic substances [14]. Solubility of phenolic compounds is governed by the type of solvent (polarity) used. The commonly used types of solvent for extracting polyphenols are methanol, ethanol, acetone, and their water solutions [75]. Thus, alcoholic solvents have been commonly employed to extract phenolics from natural sources, because they give relatively high yield of total extract, even though they are not highly selective for phenols. Particularly, mixtures of alcohols and water have revealed to be more efficient in extracting phenolic constituents than the corresponding mono-component solvent systems [76]. Using water as a solvent does not achieve high efficiency of extraction because some molecules contain sugars in their structure, which are soluble in water [75]. 

Moreover, the extraction depends on the duration of the solvent’s influence on the substrate containing phenolic compounds. It hab been reported, that extraction periods usually varying from 1 min to 24 h [77]. Longer extraction times increase the chance of oxidation of phenolics unless reducing agents are added to the solvent system [78]. Extraction temperature is one of the important factors affecting the extraction rate of polyphenols. At higher temperature, a higher yield of polyphenols extracted from fruit can be obtained. High-temperature solvents will promote polysaccharides in cell walls to distribute to solvent, and to weaken or undermine the integrity of the cell wall, thus, more polyphenols can be dissolved in the solvent used. All of these factors allow both to determine individual components and to obtain the characteristic patterns of real samples [76].

### 1.3. Analysis of polyphenols

Spectroscopy, chromatography and electrophoresis are the main methods used for polyphenols determination. Spectroscopic methods are useful only for the determination of large amounts of polyphenol compounds in a sample. Investigations devoted to this problem using HPLC with UV detection are the most numerous [75]. Although less used in analysis, since the early 1960s, thin-layer chromatography has been in vogue in phenolic analysis and still plays a distinct role in the determination of phenols in natural products [74,79]. It is especially useful for the rapid screening of plant extracts for pharmacologically active substances prior to detailed analysis. In the last twenty years, HPLC has been the analytical technique that has dominated the separation and characterisation of phenolic compounds. Due to the relatively high-molecular mass and intrinsic features of hydrophobic flavonoid aglycones and hydrophilic flavonoid glycosides, the overwhelming majority of chromatographic methods in the literature fall in the realm of HPLC and related technologies. HPLC techniques offer a unique chance to separate simultaneously all analysed components together with their possible derivatives or degradation products. In many cases, they enable the determination of low concentrations of analytes in the presence of many other interfering and coeluting components. There are many advantages dictating the widespread use of HPLC in the analysis of phenolic compounds in plant-derived and biological matrices, such as: (i) the wide range of commercially available columns, including those using new generation sorbents with fit for-purpose properties and (ii) the possibility of combining two or more columns in a switching mode [73]. 

Phenolics are commonly detected using ultraviolet/visible (UV/VIS), photodiode array (PDA), and UV-fluorescence detectors [80,81,82]. Other methods used for the detection of phenolics include electrochemical coulometric array detection, on-line connected PDA and electroarray detection, chemical reaction detection techniques, mass spectrometric and nuclear magnetic resonance detection [83,84,85]. It is evident that phenolics absorb well in the UV range and UV detection is therefore a convenient method to localise a phenol in the effluent of a column. However, no single wavelength is sufficient for their simultaneous monitoring in various natural plant extracts. Therefore, electrochemical detection provides a high selectivity and sensitivity, thus laborious sample pre-treatments such as extraction, purification or concentration are not necessary [86].

The objective of this study was to determine protocatechuic acid, 4-aminobenzoic acid, chlorogenic acid, caffeic acid, vanillin, *p*-coumaric acid, rutin, ferulic acid, quercetin, resveratrol and quercitrin from apricot using HPLC with electrochemical and UV detector and compare their sensitivity for chosen substances. Moreover, the solvent composition and influence of conditions of extraction efficiency were tested.

## 2. Results and Discussion

### 2.1. Optimization of electrochemical detection 

**Figure 2:**
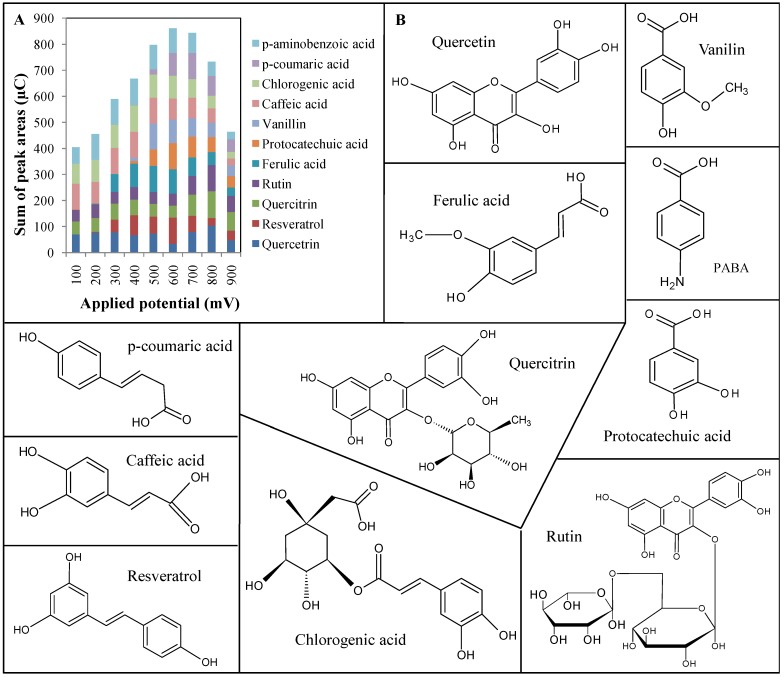
**(a)** Electrochemical response of applied potential on peak areas. Sum of peak areas show that the highest yield of extraction was at 600 mV potential. Areas of single peak of polyphenols show their extraction efficiency. Low potential did not provide protocatechuic acid detection. **(b)** Chemical structures of chosen polyphenols.

Primarily, we optimized detection potential as the fundamental parameter for HPLC-ED measurements. The applied potential of electrochemical detector was changed within the range from 100 to 900 mV. The measurement step between each potential was 100 mV. All compounds were measured under each potential three times for calculation of RSD, which was lower than 4.5%. Stock solutions of all standards (1 mL) were arranged in an autosampler and cooled at 8 °C. Analysis of each injection took 1 minute. The measured signal from the electrochemical detector represented oxidative behaviour of the single molecule under each of tested potentials. After collecting of the data we used them for various interpretations of hydrodynamic voltammograms. To select a potential for simultaneous determination of phenols, heights of signals measured under each potential were summed and are shown in Figure 2A. Behaviour of the analytes differed markedly within the tested range of potentials. Only six analytes gave a detectable signal at 100 mV. The highest signal of 4-aminobenzoic acid, chlorogenic acid, caffeic acid was detected at a potential of 400 mV, vanillin and ferulic at 500 mV, protocatechuic acid and resveratrol at 600 mV, *p*-coumaric acid at 700 mV and rutin, quercetin and quercitrin at 800 mV. Due to the highest sum of signals, the most suitable and versatile potential of detection was determined to be 600 mV. The other reason why this potential was selected is that measurement using lower potentials did not detect any signal for protocatechuic acid. This is a consequence of compromise that no substance from the group is in single potential significantly discriminated. We used this potential for determination of polyphenols in apricots. Structure of measured polyphenols is shown in Figure 2B.

### 2.2. Comparison of UV and ED 

**Figure 3:**
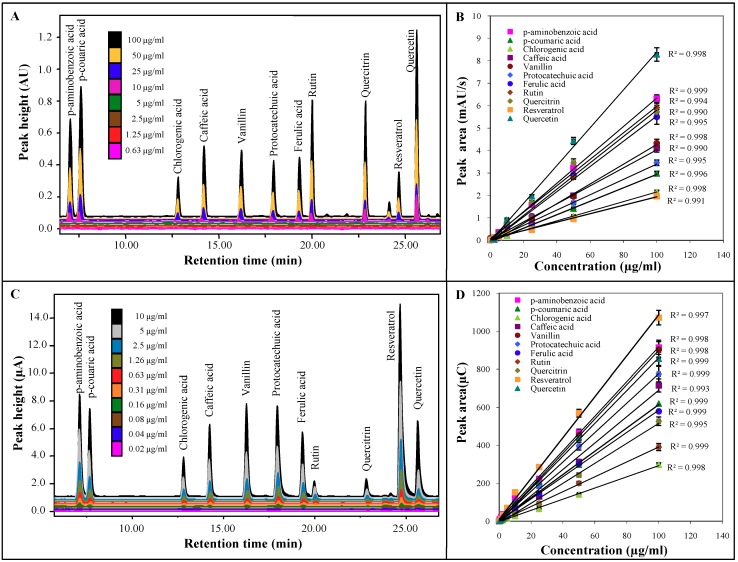
**(a)** The HPLC-UV chromatogram for determination phenolic compounds. Absorbance shows the quantity of particular substances in samples. **(b)** Dependence of peak area (mAU/s) on concentration (µg/g) and of single phenols. **(c)** The HPLC-ED chromatogram for determination of phenolic compounds. **(c)** Dependence of concentration (µg/g) and peak area (µC) of single phenols.

UV and electrochemical detector were coupled. UV detector was chosen as the first one due to the fact that it is not destructive. The highest concentration 100 µg/mL and subsequent dilution with water in ratio 1:1 of the previous concentration were prepared for constructions of calibration dependencies. The resulting analytical parameters of UV detection are shown in Table 1. Coefficients of determination of calibration curves showed good linearity. The lowest limits of detection (3 S/N) were estimated for ferulic acid (3 µM), quercitrin (4 µM) and quercetin (4 µM). Analytical parameters of electrochemical detection show a similar trend in the linearity of coefficients of determination of calibration curves. However, the detection limits are lowered by two orders of magnitude than for UV detection (Table 2). Using electrochemical detector LOD values of 27 nM, 40 nM and 37 nM were reached for ferulic acid, quercitrin and quercetin, respectively. The marked difference in the sensitivity of both detectors is evident in comparison of chromatograms of both detection techniques (Figure 3). 

The lowest limits of electrochemical detection were observed in ferulic acid, quercitrin, resveratrol and quercetin. These results indicate that the coulometric detection is under universal potential of 600 mV much suitable and sensitive for polyphenols determination than UV detection under universal wavelength 260 nm.

### 2.3. Testing of solvents for extraction of phenolics

**Figure 4:**
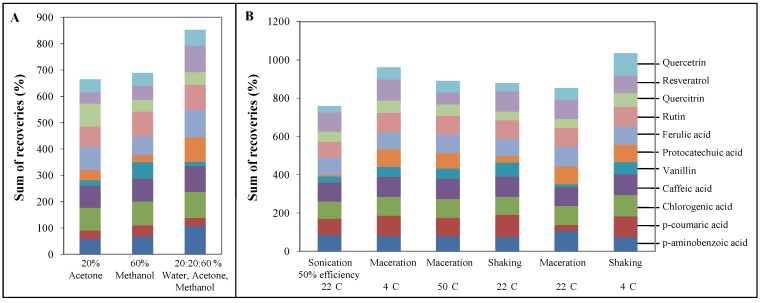
(a) Sum of recoveries for three various extraction solvents (b) Sum of recoveries for six various conditions (e.g., maceration 22 °C as control).

It is a common knowledge that the chemical extraction yield is dependent on the type and polarity of solvents, extraction time and temperature, sample-to-solvent ratio as well as on the chemical composition and physical characteristics of the sample. The solubility of phenolics is governed by the chemical nature of the plant sample, as well as the polarity of the solvents used. Plant materials may contain phenolics varying from simple to highly polymerized substances in different quantities. Therefore, there is no universal extraction solvent suitable for extraction of all plant phenolics. Solvents such as 20% acetone, 60% methanol and their combination with water in a 20:20:60% ratio (WAM) was used for the extraction of phenolics from lyophilizates of LE-1075 cultivar. This cultivar was used in our experiments because of a high content of polyphenols in comparison to other cultivars. All samples were spiked with standards to determine the recovery. For spiking low, medium and high concentrations of the polyphenols of interest were used. These were 5 μg/mL for rutin and chlorogenic acid, 0.628 μg/mL for caffeic acid, vanillin, quercetin and 0.078 μg/mL for *p*-aminobenzoic acid, protocatechuic acid, *p*-coumaric acid, ferulic acid, resveratrol and quercitrin. The recovery for the individual substances was calculated from the measured values (Table 3, Table 4 and Table 5). It is evident that the highest recovery of polyphenols was achieved using WAM solvent (Figure 4A). However, it is obvious that the effectiveness of extraction of individual substances is different for particular solvents because of different chemical structures of the polyphenols. Many authors have considered [87], aqueous methanol as one of the most effective extraction solvents. Our results showed that WAM solution in comparison with other solvents was the most effective for *p*-aminobenzoic acid, chlorgenic acid, caffeic acid, protocatechuic acid, ferulic acid, rutin, resveratrol and qercetin. Other polyphenols such as *p*-coumaric acid and vanillin had the highest recovery using a solvent of 60% methanol and quercitrin using 20% acetone. In comparison to other authors, Turkmen reached a much higher recovery of polyphenols using 50% acetone than with other solvents [88]. Generally, similar results were reached using 60% methanol and 20% acetone as a solvent but only in quercitrin analysis, the higher recovery was determined using of 20% acetone. In Figure 4 results achieved from WAM extraction are shown corresponding with the results of Krygier *et al.* [89].

### 2.4. Testing of sonication and vortexing on extraction of phenolics

Various extraction operations can influence the extraction yield. In this study, we tested the influence of sonication, vortexing and non-physico-chemical operations on extraction efficiency. To determine the effect of physical extraction methods, the above optimized MAW extraction solution was used. Lyophilized sample (LE-1075) was crushed in a mortar with the extraction solution for 2 min at 4 °C to avoid evaporation of reagents. Further, the following physical methods of extraction were tested: sonication at 50% efficiency and room temperature 22 °C, maceration at 4 °C, maceration at 50 °C, vortexing at 22 °C, maceration at 22 °C and vortexing at 4 °C. All samples were spiked with their standards to determine the recovery. For spiking low, medium and high concentrations of the polyphenols of interest were used. These were 5 μg/mL for rutin and chlorogenic acid, 0.628 μg/mL for caffeic acid, vanillin, quercetin and 0.078 μg/mL for *p*-aminobenzoic acid, protocatechuic acid, *p*-coumaric acid, ferulic acid, resveratrol and quercitrin. The recovery of the individual substances was calculated from the measured values (Table 6, Table 7, Table 8, Table 9 and Table 10). Physical methods had a greater influence on the yield of total polyphenolic compounds than the individual compounds (Figure 4B). Many authors have extracted polyphenolic substances at room and higher temperatures [90]. Our results show that for the apricot matrix we achieved a good yield of determined compounds by using maceration at 4 °C and 50 °C. All physical procedures carried out at room temperature had lower yield compared to higher temperatures due to faster diffusion. Even lower yields than the maceration at room temperature were achieved by sonication. Generally, sonication is efficient and rapid extraction method [91,92]. Using ultrasonic waves we observed relatively low yield for protocatechuic acid, vanillin and quercitrin (Figure 4B). This could be caused by partial degradation of the monitored compounds. Based on the results obtained it can be concluded that the highest yield was reached by vortexing at 4 °C. Under these conditions, the highest recoveries were obtained for all studied polyphenols except for *p*-aminobenzoic acid, which recovery was highest using maceration at 4 °C.

### 2.5. Real samples of apricots

**Figure 5:**
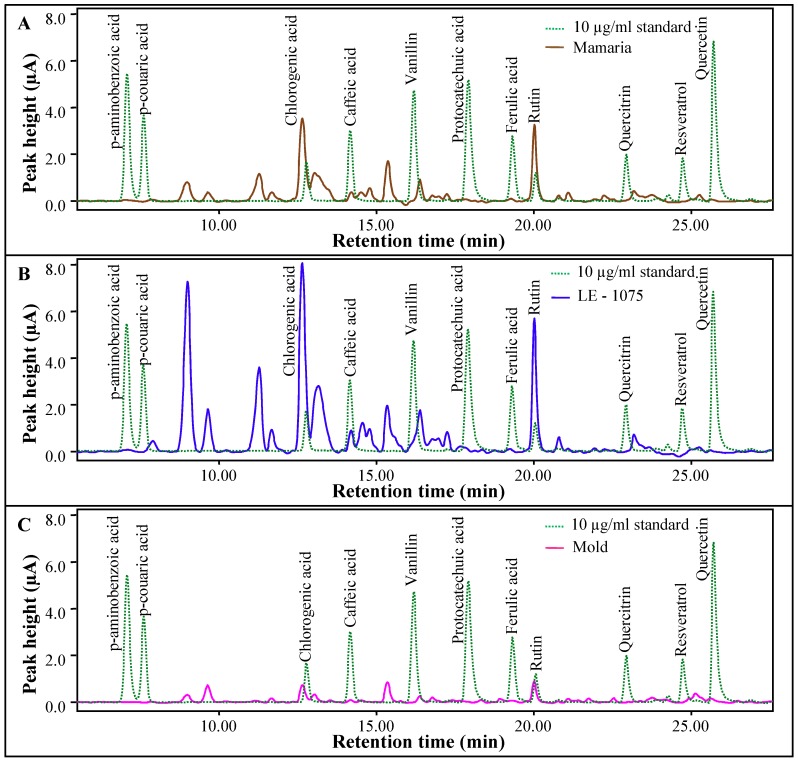
(a) Real chromatogram of Mamaria cultivar overlaid with standard mixture of analyzed polyphenols. (b) Real chromatogram of LE-1075 cultivar overlaid with standard mixture of analyzed polyphenols. (c) Real chromatogram of Mold cultivar overlaid with standard mixture of analyzed polyphenols.

Further, our optimized method was used for preparation and analysis of real samples of apricots. Cultivars Mamaria, Mold and LE-1075 were prepared using solvent mixture of 20:20:60% WAM and vortexing at 4 °C, as the optimal conditions with the highest yield. The content of each polyphenol in each cultivar varied (Figure 5, Table 11). Chlorgenic acid and rutin were the major phenolic compounds in apricot fruits. Similar results were determined also by Bors *et al.* [93]. Average content of chlorgenic acid was 2302 mg/100 g in cultivar LE-1075, 546 mg/100 g in cultivar Mamaria and 128.59 mg/100 g in cultivar Mold. According to Dragovic-Uzelac *et al.* the quantity of chlorgenic acid in apricot fruits varied depending on maturity stages but no correlation between the degree of maturity and its amount was observed [94]. Quercitrin (227 mg/100 g of apricot) and vanilin (261.89 mg/100 g of apricot) were present in remarkable amounts in cultivar LE-1075, but in other two cultivars they were negligible (0.79 mg/100 g in Mold and 1.72 mg/100 g in Mamaria). The other polyphenols identified in apricot fruits (protocatechuic acid, *p*-coumaric, *p*-aminobenzoic acid and ferulic acid) were of low content compared to the previous ones. Generally, the cultivar LE-1075 reaches the highest values in polyphenols content in contrary to Mold, which was relatively poor from the point of the content of phenolics. 

## 3. Experimental 

### 3.1. Chemicals and pH measurements

The stock standard solutions of polyphenols (1 mM) was prepared with acetonitrile (Sigma-Aldrich, USA) and stored in the dark at –20 °C. Working standard solutions were prepared daily by dilution of the stock solutions with acetonitrile. All chemical used were of ACS purity (chemicals meeting the specifications of the American Chemical Society). The pH value was measured using inoLab Level 3 with terminal Level 3 (Wissenschaftlich-Technische Werkstätten–WTW, Weilheim, Germany), controlled by the personal computer program (MultiLab Pilot; WTW). The pH-electrode (SenTix-H, pH 0–14/3M KCl) was calibrated by set of buffers (WTW). Deionised water underwent demineralization by reverse osmosis using the instruments Aqua Osmotic 02 (Aqua Osmotic, Tisnov, Czech Republic) and then it was subsequently purified using Millipore RG (Millipore Corp., USA, 18 MΏ) – MiliQ water. 

### 3.2. Biological material 

Apricots crossbreed (*Prunus armeniaca L.*) “Mold”, “Mamaria”, “LE-1075” were used in our experiments. Plants were planted in Czech Republic, in area of Lednice municipality, climatic region T4. Fruits were harvested at consumption maturity (20^th^ of July 2010). Weight of fruits on trees was 30 kg. 

### 3.3. Sample preparation

Frozen fruits were thawed at laboratory temperature and subsequently a sample of each cultivar (5 g) including flesh and peel was taken. Flesh and peel were homogenized in a mortar. The obtained homogenate was transferred into a Petri dish where it was spread for lyophilization (Christ Alpha 1-2) for 24 hours in 1^−10^ mBar and −50 °C. Subsequently, the lyophilizates were frozen with liquid nitrogen and spread in a mortar. A sample of lyophilzates (weight of 25 mg) spread for 2 minutes in the mortar with solvent (0.5 mL) at 4 °C was used for extraction. The low temperature was to prevent solvent evaporation. The samples were centrifuged (Eppendorf centrifuge 5417R) at 25,000 g, 4 °C for 30 min after the different extraction methods and subsequently, supernatant was removed and directly analyzed by HPLC.

### 3.4. HPLC measurements

The instrument for HPLC-UV-ED analysis consisted of a solvent delivery pump operating in range of 0.001–9.999 mL·min^−1^ (Model 582 ESA Inc., Chelmsford, MA, USA), chromatographic column Phenomenex Gemini C_18_ (150 × 4.6; 3 µm particles, Phenomenex, USA), UV-VIS detector (Model 528, ESA, USA) and twelve-channel CoulArray electrochemical detector (Model 5600A, ESA, USA). Detector consists of three flow analytical chambers (Model 6210, ESA, USA). Each chamber contains four analytical cells. One analytical cell contains two referent (hydrogen-palladium), and two counters and one porous graphite working electrode. Electrochemical detector is situated in control module which is thermostated. The sample (20 μL) was injected using an autosampler (Model 542, ESA, USA). The data obtained were treated by a Coularray data station (ESA, USA). The experiments were carried out at room temperature. Mobile phase consists of A: citric acid (75 mM) a B: ammonium acetate (25 mM). Flow rate was 1 mL·min^-1^. Chromatographic column was thermostated to 35 °C. Compounds were eluted by following linear increasing gradient: 0→1 min (5% B), 1→4 min (6%B), 4→20 min (25% B), 20→30 min (100% B), 30→36 min (100% B), 36→38 min (5% B), 38→45 min (5% B). Detection on UV detector was carried out at 260 nm and detection on ED detector was carried out at 600 mV. Time of one analysis was 45 minutes. 

### 3.5. Estimation of detection limit

The detection limits (3 signal/noise, S/N) were calculated according to Long and Winefordner [95], whereas N was expressed as standard deviation of noise determined in the signal domain unless stated otherwise.

## 4. Conclusions

Plant materials may contain phenolics varying from simple to highly polymerized substances in different quantities. Therefore, there is no universal method for their determination. In this work we focused on identifying a suitable method for extraction and determination of polyphenols from apricot samples. We determined protocatechuic acid, 4-aminobenzoic acid, chlorogenic acid, caffeic acid, vanillin, *p*-coumaric acid, rutin, ferulic acid, quercetin, resveratrol, quercitrin from apricot using HPLC with electrochemical and UV detector. The developed method was used for the determination of limits of detection and limits of quantification for apricot samples. Varying amounts of the chosen polyphenols was observed in the apricot cultivars Mamaria, Mold and LE-1075. The major phenolic compounds were chlorgenic acid and rutin. 

## Figures and Tables

**Table 1:** Analytical parameters of UV detection.

Compounds^1^	Regresion equation	Linear dynamic range (µM)	Linear dynamic range (µg/mL)	R^2, 2^	LOD^3^ (µM)	LOD (µg/mL)	LOD (nmol per injection)	LOQ ^4^ (µM)	LOQ (µg/mL)	LOQ (nmol per injection)	RSD^5^ (%)
*p*-Aminobenzoic acid	y = 0.0633x + 0.0085	0.918 - 609.162	0.3125–100	1.000	9	2	0.2	31	5	0.6	2.6
*p*-Coumaric acid	y = 0.03x – 0.0663	1.904– 729.182	2.5–100	0.997	23	3	0.5	77	11	1.5	4.2
Chlorogenic acid	y = 0.0215x + 0.0083	18.229–282.239	0.3125–100	0.999	13	4	0.3	42	15	0.8	3.8
Caffeic acid	y = 0.0401x + 0.0176	0.882–555.062	0.039–100	0.991	13	2	0.3	44	8	0.9	4.5
Vanillin	y = 0.0439x – 0.1269	0.216–655.824	2.5–100	0.998	14	2	0.3	47	7	0.9	4.1
Protocatechuic acid	y = 0.0341x – 0.016	16.396–648.845	0.01953–100	0.996	18	3	0.4	60	9	1.2	3.6
Ferulic acid	y = 0.0562x – 0.0818	0.127–163.795	0.625–100	0.996	3	2	0.1	9	6	0.2	6.0
Rutin	y = 0.0586x – 0.0671	1.024–514.986	0.15625–100	0.995	8	2	0.2	28	5	0.6	3.2
Quercitrin	y = 0.059x + 0.0657	0.805–223.025	0.3125–100	0.991	4	2	0.1	12	5	0.2	2.5
Resveratrol	y = 0.0188x + 0.0567	0.697–437.828	0.625–100	0.992	22	5	0.4	74	17	1.5	3.9
Quercetin	y = 0.0838x – 0.0105	2.736–330.863	0.25–100	0.998	4	1	0.1	13	4	0.3	5.2

^1^ studied flavonoid; ^2^ regression coefficients; ^3^ limits of detection of detector (3 S/N); ^4^ limits of quantification of detector (10 S/N); ^5^ relative standard deviations

**Table 2:** Analytical parameters of electrochemical detection.

Compounds^1^	Regresion equation	Linear dynamic range (µM)	Linear dynamic range (µg/mL)	R^2, 2^	LOD^3^ (µM)	LOD (µg/mL)	LOD (nmol per injection)	LOQ ^4^ (µM)	LOQ (µg/mL)	LOQ (nmol per injection)	RSD^5^ (%)
*p*-Aminobenzoic acid	y = 9.093x + 7.7544	11.59–609.16	0.01953–100	0.999	64	10	1.3	213	35	4	4.1
*p*-Coumaric acid	y = 6.125x + 1.7966	132.92–729.18	0.01953–100	0.999	114	16	2.3	378	52	8	3.3
Chlorogenic acid	y = 2.9465x – 1.5373	2.48–282.23	0.07812–100	0.999	91	32	1.8	304	108	6	3.5
Caffeic acid	y = 6.9471x – 4.8188	1.20–555.06	0.07812–100	0.994	76	14	1.5	254	46	5	4.8
Vanillin	y = 9.0092x – 1.8005	107.52–655.82	0.0048825–100	0.999	69	11	1.4	231	35	5	4.6
Protocatechuic acid	y = 5.8399x + 0.9644	0.82–648.84	0.001220625–100	0.999	106	16	2.1	353	54	7	5.2
Ferulic acid	y = 5.8399x + 0.9644	1.67–163.79	0.001220625–100	0.999	27	16	0.5	89	54	2	4.4
Rutin	y = 3.9155x – 0.2336	4.41–514.98	0.02441251–100	0.999	126	24	2.5	418	81	8	4.7
Quercitrin	y = 5.2613x – 10.4580	1.55–223.02	0.0625–100	0.995	40	18	0.8	135	60	3	4.3
Resveratrol	y = 10.759x + 10.4070	11.98–437.82	0.07812–100	0.998	39	9	0.8	129	30	3	3.6
Quercetin	y = 8.6111x – 0.7735	2.73–330.86	0.15625–100	0.999	37	11	0.7	122	37	2	4.1

^1^ studied flavonoid; ^2^ regression coefficients; ^3^ limits of detection of detector (3 S/N); ^4^ limits of quantification of detector (10 S/N); ^5^ relative standard deviations.

**Table 3:** Add Recovery of 20% Acetone.

Compounds	Homogenate (ng/mL)	Spiking (ng/mL)	Homogenate + spiking (ng/mL)	Recovery (%)
p-aminobenzoic acid	56 ± 4	495 ± 35	319 ± 22	58
p-coumaric acid	45 ± 5	131 ± 16	58 ± 7	33
Chlorogenic acid	5014 ± 301	4936 ± 296	8546 ± 513	86
Caffeic acid	128 ± 11	570 ± 51	586 ± 53	84
Vanillin	356 ± 29	651 ± 52	209 ± 17	21
Protocatechuic acid	15 ± 1	119 ± 12	53 ± 5	40
Ferulic acid	40 ± 6	76 ± 11	100 ± 14	86
Rutin	4890 ± 342	5555 ± 389	8205 ± 574	79
Quercitrin	0 ± 0	171 ± 9	150 ± 7	87
Resveratrol	48 ± 5	1173 ± 129	528 ± 58	43
Quercetin	159 ± 19	368 ± 44	259 ± 31	49

**Table 4:** Recovery of 60% Methanol.

Compounds	Homogenate (ng/mL)	Spiking (ng/mL)	Homogenate + spiking (ng/mL)	Recovery (%)
p-aminobenzoic acid	52 ± 4	411 ± 29	315 ± 22	68
p-coumaric acid	32 ± 4	122 ± 15	64 ± 8	42
Chlorogenic acid	3762 ± 226	4936 ± 296	7880 ± 473	91
Caffeic acid	41 ± 4	559 ± 50	527 ± 47	88
Vanillin	32 ± 3	654 ± 52	427 ± 34	62
Protocatechuic acid	60 ± 6	95 ± 9	40 ± 4	26
Ferulic acid	50 ± 7	76 ± 11	90 ± 13	71
Rutin	8320 ± 582	6898 ± 483	14348 ± 1004	94
Quercitrin	5 ± 0	238 ± 12	109 ± 5	45
Resveratrol	73 ± 8	1079 ± 119	622 ± 68	54
Quercetin	162 ± 19	268 ± 32	207 ± 25	48

**Table 5:** Recovery of 20:20:60% – Water, Acetone, Methanol.

Compounds	Homogenate (ng/mL)	Spiking (ng/mL)	Homogenate + spiking (ng/mL)	Recovery (%)
p-aminobenzoic acid	11 ± 1	317 ± 22	350 ± 25	107
p-coumaric acid	47 ± 6	88 ± 11	42 ± 5	31
Chlorogenic acid	12040 ± 722	4936 ±296	16964 ± 1018	100
Caffeic acid	282 ± 25	526 ± 47	790 ± 71	98
Vanillin	817 ± 65	643 ± 51	235 ± 19	16
Protocatechuic acid	15 ± 1	41 ± 4	52 ± 5	93
Ferulic acid	115 ± 16	76 ± 11	198 ± 28	104
Rutin	7388 ± 517	4924 ± 345	11921 ± 834	97
Quercitrin	0 ± 0	196 ± 10	93 ± 5	48
Resveratrol	11 ± 1	988 ± 109	1006 ± 111	101
Quercetin	129 ± 15	250 ± 30	227 ± 27	60

**Table 6:** Sonication at 22 °C, 50% efficiency.

Compounds	Homogenate (ng/mL)	Spiking (ng/mL)	Homogenate + spiking (ng/mL)	Recovery (%)
p-aminobenzoic acid	21 ± 1	325 ± 23	300 ±21	87
p-coumaric acid	18 ±2	137 ± 16	129 ± 15	83
Chlorogenic acid	11023 ± 661	4936 ± 296	14486 ± 869	91
Caffeic acid	281± 25	498 ± 45	773 ± 70	99
Vanillin	604 ± 48	565 ± 45	364 ± 29	31
Protocatechuic acid	58 ± 6	55 ± 5	10 ± 1	9
Ferulic acid	90 ± 13	76 ± 11	150 ± 21	90
Rutin	15605 ± 1092	6228 ± 436	17768 ± 1244	81
Quercitrin	1 ± 0	179 ± 9	97 ± 5	54
Resveratrol	2 ± 0	965 ± 106	963 ± 106	100
Quercetin	135 ± 16	224 ± 27	120 ± 14	33

**Table 7:** Maceration 4 °C.

Compounds	Homogenate (ng/mL)	Spiking (ng/mL)	Homogenate + spiking (ng/mL)	Recovery (%)
p-aminobenzoic acid	11 ± 1	262 ± 18	218 ± 15	80
p-coumaric acid	10 ± 1	121 ± 14	139 ± 17	106
Chlorogenic acid	12666 ± 760	4936 ± 296	17420 ± 1045	99
Caffeic acid	276 ± 25	415 ± 37	719 ± 65	104
Vanillin	190 ± 15	472 ± 38	348 ± 28	53
Protocatechuic acid	10 ± 1	47 ± 5	54 ± 5	93
Ferulic acid	103 ± 14	76 ± 11	158 ± 22	88
Rutin	15710 ± 1100	5354 ± 375	21093 ± 1477	100
Quercitrin	2 ± 0	142 ± 7	95 ± 5	66
Resveratrol	9 ± 1	803 ± 88	884 ± 97	109
Quercetin	101 ± 12	200 ± 24	190 ± 23	63

**Table 8:** Maceration 50 °C.

Compounds	Homogenate (ng/mL)	Spiking (ng/mL)	Homogenate + spiking (ng/mL)	Recovery (%)
p-aminobenzoic acid	35 ± 2	262 ± 18	238 ± 17	80
p-coumaric acid	22 ± 3	113 ± 14	129 ± 16	96
Chlorogenic acid	15357 ± 921	4936 ± 296	19812 ± 1189	98
Caffeic acid	328 ± 30	408 ± 37	775 ± 70	105
Vanillin	257 ± 21	462 ± 37	393 ± 31	55
Protocatechuic acid	17 ± 2	48 ± 5	52 ± 5	80
Ferulic acid	122 ± 17	76 ± 11	192 ± 27	97
Rutin	19300 ± 1351	5464 ± 382	24366 ± 1706	98
Quercitrin	0 ± 0	141 ± 7	83 ± 4	59
Resveratrol	39 ± 4	805 ± 89	533 ± 59	63
Quercetin	122 ± 15	195 ± 23	192 ± 23	61

**Table 9:** Shaking 22 °C.

Compounds	Homogenate (ng/mL)	Spiking (ng/mL)	Homogenate + spiking (ng/mL)	Recovery (%)
p-aminobenzoic acid	19 ± 1	290 ± 20	290 ± 20	76
p-coumaric acid	10 ± 1	77 ± 9	77 ± 9	115
Chlorogenic acid	11056 ± 663	4936 ± 296	4936 ±296	94
Caffeic acid	237 ± 21	442 ± 40	442 ± 40	107
Vanillin	886 ± 71	477 ± 38	477 ± 38	73
Protocatechuic acid	25 ± 2	142 ± 14	142 ± 14	34
Ferulic acid	100 ± 14	76 ± 11	76 ± 11	88
Rutin	14316 ± 1002	4993 ± 349	4993 ± 349	98
Quercitrin	1 ± 0	156 ± 8	156 ± 8	45
Resveratrol	41 ± 5	869 ± 96	869 ± 96	105
Quercetin	78 ± 9	146 ± 18	146 ± 18	43

**Table 10:** Shaking 4 °C.

Compounds	Homogenate (ng/mL)	Spiking (ng/mL)	Homogenate + spiking (ng/mL)	Recovery (%)
p-aminobenzoic acid	155 ± 11	2589 ± 181	2033 ± 142	74
p-coumaric acid	14 ± 2	943 ± 113	1039 ± 125	109
Chlorogenic acid	13397 ± 804	2613 ± 157	17969 ± 1078	112
Caffeic acid	338 ± 30	629 ± 57	1043 ± 94	108
Vanillin	8 ± 1	613 ± 49	396 ± 32	64
Protocatechuic acid	89 ± 9	1655 ± 166	1572 ± 157	90
Ferulic acid	104 ± 15	619 ± 87	687 ± 96	95
Rutin	10646 ± 745	3256 ± 228	14276 ± 999	103
Quercitrin	82 ± 4	253 ± 13	241 ± 12	72
Resveratrol	83 ± 9	191 ± 21	254 ± 28	93
Quercetin	164 ± 20	406 ± 49	666 ±80	117

**Table 11:** Analytical results.

Compounds	Mold (ng/mL)	LE-1075 (ng/mL)	Mamaria (ng/mL)
p-aminobenzoic acid	100 ± 7	501 ± 35	01,427 ± 99
p-coumaric acid	1,722 ± 155	20,49 ± 1,844	129 ± 11
Chlorogenic acid	64,294 ± 8,358	1,150,979 ± 149,627	273,019 ± 35,492
Caffeic acid	2,937 ± 176	16,663 ± 999	7,042 ± 422
Vanillin	15,664 ± 2,193	130,946 ± 18,332	805 ± 112
Protocatechuic acid	1,117 ± 122	959 ± 105	2,040 ± 224
Ferulic acid	3,267 ± 294	8,460 ± 761	6,369 ± 573
Rutin	60,457 ± 3,022	373,734 ± 18,686	346,355 ± 17,317
Quercitrin	396 ± 39	113,500 ± 11,350	861 ± 86
Resveratrol	96 ± 12	2,256 ± 293	136 ± 17
Quercetin	3,611 ± 541	3,241 ± 486	2,772 ± 415

## References

[1] Bruneton J. (1999). Pharmacognosy, Phytochemistry, Medicinal Plants.

[2] Cartea M.E., Francisco M., Soengas P., Velasco P. (2010). Phenolic Compounds in Brassica Vegetables. Molecules.

[3] Ferretti G., Bacchetti T., Belleggia A., Neri D. (2010). Cherry Antioxidants: From Farm to Table. Molecules.

[4] Sochor J., Zitka O., Skutkova H., Pavlik D., Babula P., Krska B., Horna A., Adam V., Provaznik I., Kizek R. (2010). Content of Phenolic Compounds and Antioxidant Capacity in Fruits of Apricot Genotypes. Molecules.

[5] Sisa M., Bonnet S.L., Ferreira D., Van der Westhuizen J.H. (2010). Photochemistry of Flavonoids. Molecules.

[6] Ghasemzadeh A., Jaafar H.Z.E., Rahmat A. (2010). Elevated Carbon Dioxide Increases Contents of Flavonoids and Phenolic Compounds, and Antioxidant Activities in Malaysian Young Ginger (Zingiber officinale Roscoe.) Varieties. Molecules.

[7] Kelsey N.A., Wilkins H.M., Linseman D.A. (2010). Nutraceutical Antioxidants as Novel Neuroprotective Agents. Molecules.

[8] Rechner A.R., Wagner E., Van Buren L., Van de Put F., Wiseman S., Rice-Evans C.A. (2002). Black tea represents a major source of dietary phenolics among regular tea drinkers. Free Radic. Res..

[9] Gonzalez-Gallego J., Garcia-Mediavilla M.V., Sanchez-Campos S., Tunon M.J. (2010). Fruit polyphenols, immunity and inflammation. Br. J. Nutr..

[10] Galleano M., Pechanova O., Fraga C.G. (2010). Hypertension, Nitric Oxide, Oxidants, and Dietary Plant Polyphenols. Curr. Pharm. Biotechnol..

[11] Fang Z.X., Bhandari B. (2010). Encapsulation of polyphenols - a review. Trends Food Sci. Technol..

[12] Michalowicz J., Duda W., Pol J. (2007). Environ. Stud. Pol. J. Environ. Stud..

[13] Chen H.L., Yao J., Wang F., Zhou Y., Chen K., Zhuang R.S., Choi M.M.F., Zaray G. (2010). Toxicity of three phenolic compounds and their mixtures on the gram-positive bacteria Bacillus subtilis in the aquatic environment. Sci. Total Environ..

[14] Shadnia H., Wright J.S. (2008). Understanding the toxicity of phenols: Using quantitative structure-activity relationship and enthalpy changes to discriminate between possible mechanisms. Chem. Res. Toxicol..

[15] Lepoittevin J.P., Benezra C. (1991). Allergic contact-dermatitis caused by naturally-occurring quinones. Pharm. Weekblad-Sci. Ed..

[16] Saito S., Kawabata J. (2005). Effects of electron-withdrawing substituents on DPPH radical scavenging reactions of protocatechuic acid and its analogues in alcoholic solvents. Tetrahedron.

[17] Hatzipanayioti D., Karaliota A., Kamariotaki M., Aletras V., Petropouleas P. (2006). Theoretical and spectroscopic investigation of the oxidation and degradation of protocatechuic acid. Chem. Phys..

[18] Kampa M., Alexaki V.I., Notas G., Nifli A.P., Nistikaki A., Hatzoglou A., Bakogeorgou E., Kouimtzoglou E., Blekas G., Boskou D., Gravanis A., Castanas E. (2004). Antiproliferative and apoptotic effects of selective phenolic acids on T47D human breast cancer cells: potential mechanisms of action. Breast Cancer Res..

[19] Ueda J.I., Saito N., Shimazu Y., Ozawa T. (1996). A comparison of scavenging abilities of antioxidants against hydroxyl radicals. Arch. Biochem. Biophys..

[20] An L.J., Guan S., Shi G.F., Bao Y.M., Duan Y.L., Jiang B. (2006). Protocatechuic acid from Alpinia oxyphylla against MPP+-induced neurotoxicity in PC12 cells. Food Chem. Toxicol..

[21] Akberova S.I. (2002). New biological properties of p-aminobenzoic acid. Biol. Bull..

[22] Shuang S.M., Yang Y., Pan J.H. (2002). Study on molecular recognition of para-aminobenzoic acid species by alpha-, beta- and hydroxypropyl-beta-cyclodextrin. Anal. Chim. Acta.

[23] Schmidt T.C., Petersmann M., Kaminski L., vonLow E., Stork G. (1997). Analysis of aminobenzoic acids in waste water from a former ammunition plant with HPLC and combined diode array and fluorescence detection. Fres. J. Anal. Chem..

[24] Clifford M.N. (1999). Chlorogenic acids and other cinnamates - nature, occurrence and dietary burden. J. Sci. Food Agric..

[25] Boerjan W., Ralph J., Baucher M. (2003). Lignin biosynthesis. Annu. Rev. Plant Biol..

[26] Kono Y., Kashine S., Yoneyama T., Sakamoto Y., Matsui Y., Shibata H. (1998). Iron chelation by chlorogenic acid as a natural antioxidant. Biosci. Biotechnol. Biochem..

[27] Halliwell B., Gutteridge J.M.C. (1990). Role of free-radicals and catalytic metal-ions in human-disease - an overview. Methods Enzymol..

[28] Mori H., Tanaka T., Shima H., Asu T.K., Takahashi M. (1986). Inhibitory effect of chlorogenic acid on methylazoxymethanol acetate-induced carcinogenesis in large-intestine and liver of hamsters. Cancer Lett..

[29] Tsuchiya T., Suzuki O., Igarashi K. (1996). Protective effects of chlorogenic acid on paraquat-induced oxidative stress in rats. Biosci. Biotechnol. Biochem..

[30] Zhao Z.H., Moghadasian M.H. (2010). Bioavailability of hydroxycinnamates: a brief review of in vivo and *in vitro* studies. Phytochem. Rev..

[31] Maurya D.K., Devasagayam T.P.A. (2010). Antioxidant and prooxidant nature of hydroxycinnamic acid derivatives ferulic and caffeic acids. Food Chem. Toxicol..

[32] Kono Y., Shibata H., Kodama Y., Sawa Y. (1995). The suppression of the N-nitrosating reaction by chlorogenic acid. Biochem. J..

[33] Kasai H., Fukada S., Yamaizumi Z., Sugie S., Mori H. (2000). Action of chlorogenic acid in vegetables and fruits as an inhibitor of 8-hydroxydeoxyguanosine formation in vitro and in a rat carcinogenesis model. Food Chem. Toxicol..

[34] Shibata H., Sakamoto Y., Oka M., Kono Y. (1999). Natural antioxidant, chlorogenic acid, protects against DNA breakage caused by monochloramine. Biosci. Biotechnol. Biochem..

[35] Akagi K., Hirose M., Hoshiya T., Mizoguchi Y., Ito N., Shirai T. (1995). Modulating effects of ellagic acid, vanillin and quercetin in a rat medium-term multiorgan carcinogenesis model. Cancer Lett..

[36] Kappachery S., Paul D., Yoon J., Kweon J.H. (2010). Vanillin, a potential agent to prevent biofouling of reverse osmosis membrane. Biofouling.

[37] Kumar S.S., Ghosh A., Devasagayam T.P.A., Chauhan P.S. (2000). Effect of vanillin on methylene blue plus light-induced single-strand breaks in plasmid pBR322 DNA. Mutat. Res. Genet. Toxicol. Environ. Mutagen..

[38] Aruoma O.I., Evans P.J., Kaur H., Sutcliffe L., Halliwell B. (1990). An evaluation of the antioxidant and potential pro-oxidant properties of food-additives and of trolox-c, vitamin-e and probucol. Free Rad. Res. Commun..

[39] Utsumi H., Fujii K., Irie H., Furusaki A., Nitta I. (1967). Crystal structure of p-coumaric acid. Bull. Chem. Soc. Jpn..

[40] Castelluccio C., Paganga G., Melikian N., Bolwell G.P., Pridham J., Sampson J., Riceevans C. (1995). Antioxidant potential of intermediates in phenylpropanoid metabolism in higher-plants. FEBS Lett..

[41] Sharma R.D. (1979). Isoflavones and hypercholesterolemia in rats. Lipids.

[42] Gaberscik A., Voncina M., Trost T., Germ M., Bjorn L.O. (2002). Growth and production of buckwheat (Fagopyrum esculentum) treated with reduced, ambient, and enhanced UV-B radiation. J. Photochem. Photobiol. B-Biol..

[43] Rozema J., Bjorn L.O., Bornman J.F., Gaberscik A., Hader D.P., Trost T., Germ M., Klisch M., Groniger A., Sinha R.P., Lebert M., He Y.Y., Buffoni-Hall R., de Bakker N.V.J., van de Staaij J., Meijkamp B.B. (2002). The role of UV-B radiation in aquatic and terrestrial ecosystems - an experimental and functional analysis of the evolution of UV-absorbing compounds. J. Photochem. Photobiol. B-Biol..

[44] Korkmaz A., Kolankaya D. (2010). Protective Effect of Rutin on the Ischemia/Reperfusion Induced Damage in Rat Kidney. J. Surg. Res..

[45] Abeywardena M.Y., Head R.J. (2001). Dietary polyunsaturated fatty acid and antioxidant modulation of vascular dysfunction in the spontaneously hypertensive rat. Prostagland. Leuk. Essent. Fatty Acids.

[46] Wojcicki J., Barcewwiszniewska B., Samochowiec L., Rozewicka L. (1995). Extractum-fagopyri reduces atherosclerosis in high-fat diet fed rabbits. Pharmazie.

[47] Bingjiang L., Wei M., Dan L. (2010). Photoprotective effects of ferulic on human keratinocyte HaCaT cells: Proteomic identification of proteins associated with cutaneous cancer. J. Invest. Dermatol..

[48] Zhang L.W., Al-Suwayeh S.A., Hsieh P.W., Fang J.Y. (2010). A comparison of skin delivery of ferulic acid and its derivatives: Evaluation of their efficacy and safety. Int. J. Pharm..

[49] Yabe T., Hirahara H., Harada N., Ito N., Nagai T., Sanagi T., Yamada H. (2010). Ferulic acid induces neural progenitor cell proliferation *in vitro* and *in vivo*. Neuroscience.

[50] de Boer V.C.J., Dihal A.A., van der Woude H., Arts I.C.W., Wolffram S., Alink G.M., Rietjens I., Keijer J., Hollman P.C.H. (2005). Tissue distribution of quercetin in rats and pigs. J. Nutr..

[51] Cushnie T.P.T., Lamb A.J. (2005). Antimicrobial activity of flavonoids. Int. J. Antimicrob. Agents.

[52] Seufi A.M., Ibrahim S.S., Elmaghraby T.K., Hafez E.E. (2009). Preventive effect of the flavonoid, quercetin, on hepatic cancer in rats via oxidant/antioxidant activity: Molecular and histological evidences. J. Exp. Clin. Cancer Res..

[53] Kaindl U., Eyberg I., Rohr-Udilova N., Heinzle C., Marian B. (2008). The dietary antioxidants resveratrol and quercetin protect cells from exogenous pro-oxidative damage. Food Chem. Toxicol..

[54] Orsolic N., Knezevic A.H., Sver L., Terzic S., Basic I. (2004). Immunomodulatory and antimetastatic action of propolis and related polyphenolic compounds. J. Ethnopharmacol..

[55] Arts I.C.W., Hollman P.C.H. (2005). Polyphenols and disease risk in epidemiologic studies. Am. J. Clin. Nutr..

[56] Knekt P., Kumpulainen J., Jarvinen R., Rissanen H., Heliovaara M., Reunanen A., Hakulinen T., Aromaa A. (2002). Flavonoid intake and risk of chronic diseases. Am. J. Clin. Nutr..

[57] Benkovic V., Kopjar N., Knezevic A.H., Dikic D., Basic I., Ramic S., Viculin T., Knezevic F., Orsolic N. (2008). Evaluation of radioprotective effects of propolis and quercetin on human white blood cells in vitro. Biol. Pharm. Bull..

[58] Rahman M.M., Bak I., Das D.K. (2010). Effectiveness of Resveratrol Against Cardiovascular Disease. Mini-Rev. Org. Chem..

[59] Toklu H.Z., Sehirli O., Ersahin M., Suleymanoglu S., Yiginer O., Emekli-Alturfan E., Yarat A., Yegen B.C., Senser G. (2010). Resveratrol improves cardiovascular function and reduces oxidative organ damage in the renal, cardiovascular and cerebral tissues of two-kidney, one-clip hypertensive rats. J. Pharm. Pharmacol..

[60] Chicoine L.G., Stewart J.A., Lucchesi P.A. (2009). Is Resveratrol the Magic Bullet for Pulmonary Hypertension?. Hypertension.

[61] Tiwari V., Sharma S., Kulkarni S.K., Chopra K. (2008). Amelioration of oxidative stress and renal dysfunction by insulin and its combination with curcumin or resveratrol: Role of TGF-beta. Indian J. Pharmacol..

[62] Thandapilly S.J., Wojciechowski P., Behbahani J., Louis X.L., Yu L.P., Juric D., Kopilas M.A., Anderson H.D., Netticadan T. (2010). Resveratrol Prevents the Development of Pathological Cardiac Hypertrophy and Contractile Dysfunction in the SHR Without Lowering Blood Pressure. Am. J. Hypertens..

[63] Khalil A., Berrougui H. (2009). Mechanism of action of resveratrol in lipid metabolism and atherosclerosis. Clin. Lipidol..

[64] Kaeberlein M. (2010). Resveratrol and rapamycin: are they anti-aging drugs?. Bioessays.

[65] Wagner C., Fachinetto R., Corte C.L.D., Brito V.B., Severo D., Dias G., Morel A.F., Nogueira C.W., Rocha J.B.T. (2006). Quercitrin, a glycoside form of quercetin, prevents lipid peroxidation in vitro. Brain Res..

[66] Jung M., Park M. (2007). Acetylcholinesterase inhibition by flavonoids from agrimonia pilosa. Molecules.

[67] Materska M., Perucka I. (2005). Antioxidant activity of the main phenolic compounds isolated from hot pepper fruit (Capsicum annuum L.). J. Agric. Food Chem..

[68] Davis R.A., Simpson M.M., Nugent R.B., Carroll A.R., Avery V.M., Rali T., Chen H., Qurallo B., Quinn R.J. (2008). Pim2 inhibitors from the Papua New Guinean plant Cupaniopsis macropetala. J. Nat. Prod..

[69] Ibrahim N.A., El-Seedi H.R., Mohammed M.M.D. (2007). Phytochemical investigation and hepatoprotective activity of Cupressus sempervirens L. leaves growing in Egypt. Nat. Prod. Res..

[70] Liu Y., Murakami N., Ji H., Abreu P., Zhang S. (2007). Antimalarial flavonol glycosides from Euphorbia hirta. Pharm. Biol..

[71] Fukai T., Sakagami H., Toguchi M., Takayama F., Iwakura I., Atsumi T., Ueha T., Nakashima H., Nomura T. (2000). Cytotoxic activity of low molecular weight polyphenols against human oral tumor cell lines. Anticancer Res..

[72] Dai J., Mumper R.J. (2010). Plant Phenolics: Extraction, Analysis and Their Antioxidant and Anticancer Properties. Molecules.

[73] Naczk M., Shahidi F. (2004). Extraction and analysis of phenolics in food. J. Chromatogr. A.

[74] Stalikas C.D. (2007). Extraction, separation, and detection methods for phenolic acids and flavonoids. J. Sep. Sci..

[75] Kartsova L.A., Alekseeva A.V. (2008). Chromatographic and Electrophoretic Methods for Determining Polyphenol Compounds. J. Anal. Chem..

[76] Yang L., Jiang J.G., Li W.F., Chen J., Wang D.Y., Zhu L. (2009). Optimum extraction Process of polyphenols from the bark of Phyllanthus emblica L. based on the response surface methodology. J. Sep. Sci..

[77] Cork S.J., Krockenberger A.K. (1991). Methods and pitfalls of extracting condensed tannins and other phenolics from plants - insights from investigations on eucalyptus leaves. J. Chem. Ecol..

[78] Khanna S.K., Viswanat P.N., Krishnan P.S., Sanwal G.G. (1968). Extraction of total phenolics in presence of reducing agents. Phytochemistry.

[79] Ragazzi E., Veronese G. (1973). Quantitative-analysis of phenolic compounds after thin-layer chromatographic separation. J. Chromatogr..

[80] Rodriguez-Arcos R.C., Smith A.C., Waldron K.W. (2002). Effect of storage on wall-bound phenolics in green asparagus. J. Agric. Food Chem..

[81] Barroso C.G., Rodriguez M.C., Guillen D.A., PerezBustamante J.A. (1996). Analysis of low molecular mass phenolic compounds, furfural and 5-hydroxymethylfurfural in Brandy de Jerez by high-performance liquid chromatography diode array detection with direct injection. J. Chromatogr. A.

[82] Dekic S., Milosavljevic S., Vajs V., Jovic S., Petrovic A., Nikicevic N., Manojlovic V., Nedovic V., Tesevic V. (2008). Trans- and cis-resveratrol concentration in wines produced in Serbia. J. Serb. Chem. Soc..

[83] Kivilompolo M., Oburka V., Hyotylainen T. (2008). Comprehensive two-dimensional liquid chromatography in the analysis of antioxidant phenolic compounds in wines and juices. Anal. Bioanal. Chem..

[84] Benova B., Hajek T., Kaljurand M. (2010). Utilization of coulometric array detection in analysis of beverages and plant extracts. 5th Symposium by Nordic Separation Science Society.

[85] Krafczyk N., Glomb M.A. (2008). Characterization of phenolic compounds in rooibos tea. J. Agric. Food Chem..

[86] Kahoun D., Rezkova S., Veskrnova K., Kralovsky J., Holcapek M. (2008). Determination of phenolic compounds and hydroxymethylfurfural in meads using high performance liquid chromatography with coulometric-array and UV detection. J. Chromatogr. A.

[87] Jouki M., Khazaei N., Anninos P., Rossi M., Pham T.D., Falugi C., Bussing A., Koukkou M. (2010). Compare of extraction of phenolic compounds from Pistacia atlantica in different solvents. Advances in Biomedical Research, Proceedings.

[88] Turkmen N., Velioglu Y.S., Sari F., Polat G. (2007). Effect of extraction conditions on measured total polyphenol contents and antioxidant and antibacterial activities of black tea. Molecules.

[89] Krygier K., Sosulski F., Hogge L. (1982). Free, esterified, and insoluble-bound phenolic-acids.1. Extraction and purification procedure. J. Agric. Food Chem..

[90] Rababah T.M., Banat F., Rababah A., Ereifej K., Yang W. (2010). Optimization of Extraction Conditions of Total Phenolics, Antioxidant Activities, and Anthocyanin of Oregano, Thyme, Terebinth, and Pomegranate. J. Food Sci..

[91] Rodrigues S., Pinto G.A.S., Fernandes F.A.N. (2008). Optimization of ultrasound extraction of phenolic compounds from coconut (Cocos nucifera) shell powder by response surface methodology. Ultrason. Sonochem..

[92] Gribova N.Y., Filippenko T.A., Nikolaevskii A.N., Belaya N.I., Tsybulenko A.A. (2008). Optimization of Conditions for the Extraction of Antioxidants from Solid Parts of Medicinal Plants. J. Anal. Chem..

[93] Bors W., Michel C., Das D.K., Ursini F. (2002). Chemistry of the antioxidant effect of polyphenols. Alcohol and Wine Health and Disease.

[94] Dragovic-Uzelac V., Levaj B., Mrkic V., Bursac D., Boras M. (2007). The content of polyphenols and carotenoids in three apricot cultivars depending on stage of maturity and geographical region. Food Chem..

[95] Long G.L., Winefordner J.D. (1983). Limit of Detection. Anal. Chem..

